# Improving Orthopedic Documentation Using Post-Operative Note Proformas: A Quality Improvement Study

**DOI:** 10.7759/cureus.42295

**Published:** 2023-07-22

**Authors:** Christopher McKee, Conor Brines, Scarlett O'Brien, Robert Espey, Danny Acton

**Affiliations:** 1 Trauma and Orthopaedics, Altnagelvin Area Hospital, Londonderry, GBR; 2 Orthopaedic Surgery, Belfast Health and Social Care Trust, Belfast, GBR

**Keywords:** orthopaedic postoperative instructions, patient safety improvement, post-operative documentation, quality improvement (qi), orthopaedic surgery

## Abstract

Introduction: Accurate medical documentation is important in the perioperative period, ensuring the safe transfer of information between teams involved in the surgical patient’s care. This has been highlighted by multiple standards of care guidelines within the United Kingdom. The use of standardized pre-templated documents has displayed significant success in minimizing errors during the admission and operative stages. The aim of this study is to evaluate whether a similar proforma for the post-operative stage is successful in orthopedic patients.

Methods: A retrospective review of 25 consecutive orthopedic elective patients was conducted during the first cycle. Exclusion criteria included patients who were under 16, day case procedures, and admission due to trauma. The second cycle consisted of a prospective review of 25 patients a month following the implementation of the new proforma. Both cycles were scored against 10 inclusion parameters as outlined by national guidelines.

Results: Implementation of the proforma resulted in a significant improvement in post-operative note compliance. A total of six parameters showed a statistically significant improvement (p<0.05). This included wound assessment (58.3%-100%, p<0.001), post-operative imaging (37.5%-92%, p<0.001), neurovascular assessment (83.3%-100%, p=0.017), National Early Warning Score (25.0%-100%, p<0.001), venous thromboembolism prophylaxis (29.2%-96.0%, p<0.001), and antibiotic administration (4.2%-84.0%, p<0.001).

Conclusions: Monitoring of important clinical parameters significantly improved following the implementation of the post-operative proforma. These results will hopefully cause the introduction of other proformas in other surgical specialties and other units.

## Introduction

Precise written documentation is vital in the transfer of medical information, from a patient’s initial presentation to discharge. This has become increasingly more important due to the significant increase in both medical errors and clinical negligence claims because of inadequate documentation [1]. A direct comparison between 2009 and 2019 demonstrated that orthopedics saw the greatest number of negligence claims as well as the greatest increase in claims [2]. Further analysis of the period between 2008/2009 and 2018/2019 showed that clinical negligence claims doubled (427-840). Of these 1,166 were due to parameters that should be recorded in post-operative notes, resulting in £149,504,268 in damages paid [2-3]. To minimize errors and maintain a high standard of care, multiple guidelines and standards have been implemented [4-6].

Orthopedic teams across the UK and Ireland have led initiatives to improve both admission documentation as well as post-operative in orthopedic patients. Arguments for standardized proformas prove these systems improve time efficiency and increase compliance with post-operative documentation guidelines [7-8]. Increased medical staff turnover, with most rotating every four months, has also shown no hindrance to the results of post-op proformas for elective lower limb arthroplasty patients previously [9].

This study aimed to evaluate current post-operative documentation for elective orthopedic procedures, specifically, assessing adherence to national guidelines. The appraisal of documented clinical examination and continuity of medical management was compared to that following implementation of a note proforma. Information from this study will provide insight into whether this type of proforma is beneficial in post-operative continuation of care and improvement of patient outcomes.

## Materials and methods

A quality improvement project (QIP) was initiated to improve post-operative ward round documentation of elective orthopedic patients on the day following their procedure. A retrospective review was completed of day-one ward round notes of 25 orthopedic patients in the trauma & orthopedic department of a large district general hospital. Data were collected from randomly selected patients' notes from both elective patients admitted within three weeks of the initial data collection period. Notes were arbitrarily selected from the elective notes archive, by means of stratified sampling from each consultant surgeon's list of patients. Patients were substratified based on the type of surgery, to ensure both upper limb and lower limb patients were included. The primary outcome was the assessment for compliance of the inclusion of 10 pre-determined parameters. These parameters were selected by reviewing standards of documentation in the national guidelines: Getting It Right First Time [4], Professional Records Standards Body [5], British Orthopaedic Association Guidelines [6], and Royal College of Surgeons - Good Surgical Practice [10]. The parameters consisted of date, orthopedic procedure, National Early Warning Score (NEWS), examination, wound assessment, venous thromboembolism (VTE) prophylaxis, imaging, antibiotics, blood samples, and management plan. Inclusion criteria were elective adult patients admitted under the orthopedic team, both upper and lower limb surgical patients with a hospital stay >1 day. Exclusion criteria were day case operations, acute trauma patients, and patients less than 16 years of age. 

**Figure 1:**
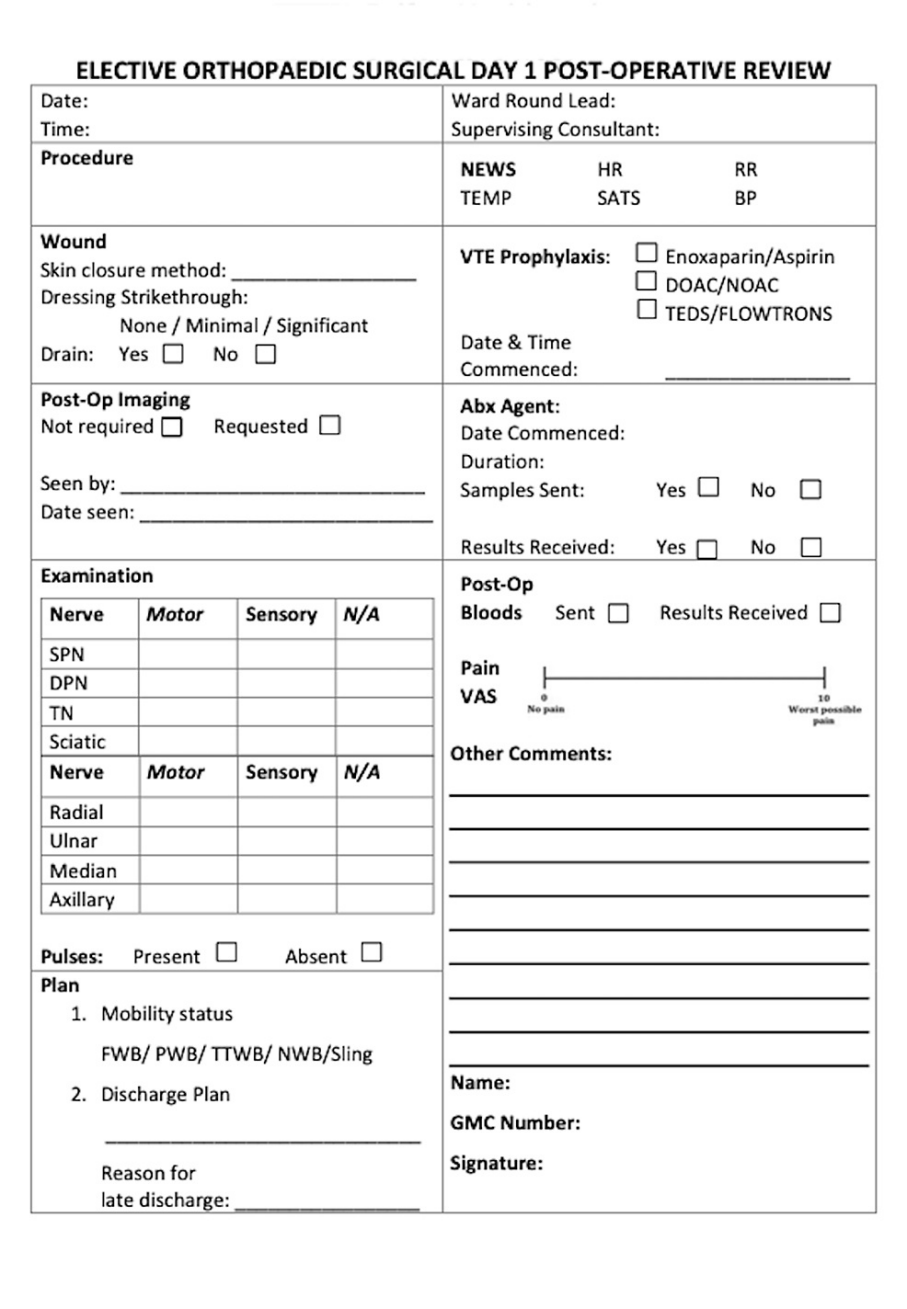
Post-operative proforma.

Review results were presented at the departmental level to members of the orthopedic multidisciplinary team (MDT). A standardized day-one post-operative ward round proforma for elective orthopedic patients at this unit was developed with the assistance of the MDT. This proforma (Figure 1) was standardized across all orthopedic wards in this institution, and designed with national clinical guidelines at its core. Proformas were included in patient’s packs making them a part of the surgery documentation bundle that accompanies the patient on their pathway. The proforma was placed after the operation note, to aid ward round flow and increase compliance during busy ward rounds. Interventions were planned and implemented in accordance with the Plan-Do-Study-Act (PDSA) improvement model. The proforma was highlighted at MDT meetings and compliance of usage was assessed on a weekly basis following implementation.

The second PDSA cycle entailed randomized data collection over a three-week period, a month following the implementation of the proforma. Results were obtained from a total of 25 medical notes and assessed compliance against the 10 parameters mentioned above. Notes were considered compliant to each parameter if it was documented in the post-operative notes during the first cycle, or the relevant section completed on the proforma during the second cycle. The Proforma usage rate was measured by assessing all available elective post-operative notes at each data collection time point. 

Microsoft Excel (Microsoft® Corp., Redmond, WA) was used for collation, and Analysis ToolPak (Microsoft® Corp., Redmond, WA) frequentist statistical analysis of results, which included figures of mean values and Fisher’s Exact Test. A p-value of <0.05 was used as a test of statistical significance. The SQUIRE 2.0 checklist for quality improvement studies was used as a framework for this study [11]. Patients or the public were not involved in the design, conduct, reporting, or dissemination plans of our research.

## Results

A total of 50 notes were assessed over two PDSA cycles between December 2022 and May 2023. The first PDSA cycle showed poor compliance across multiple current guideline parameters. Parameters with the poorest compliance included antibiotic administration (4.2%), NEWS score documented (25.0%), VTE prophylaxis (29.2%), and check imaging action (37.5%). This prompted the immediate implementation of a pre-templated proforma created following discussions with the orthopedic MDT. This implementation resulted in a significant improvement in compliance as shown in Table 1.

**Table 1:** Results of pre- vs post-implementation cycles of standardized ward round proforma for elective day-one post-operative patients. NEWS, National Early Warning Score; VTE, venous thromboembolism

	Pre-implementation compliance (%)	Post-implementation compliance (%)	p-Value
Date	100	100	N/A
Procedure	95.8	100	0.156
Wound assessment	58.3	100	<0.001
Imaging action recorded	37.5	92.0	<0.001
Neurovascular assessment	83.3	100	0.017
NEWS	25.0	92.0	<0.001
VTE prophylaxis	29.2	96.0	<0.001
Antibiotics	4.2	84.0	<0.001
Blood request/action	66.7	84.0	0.083
Management plan	100	100	N/A

From baseline to PDSA cycle 2, there was a mean increase in compliance of 34.7% across all 10 parameters (p<0.001). The greatest increase in compliance was shown in the documentation of antibiotic administration (4.2%-84.0%, p<0.001). A total of six parameters showed a statistically significant improvement. This included wound assessment (58.3%-100%, p<0.001), post-operative imaging (37.5%-92%, p<0.001), neurovascular assessment (83.3%-100%, p=0.017), National Early Warning Score (25.0%-100%, p<0.001), venous thromboembolism prophylaxis (29.2%-96.0%, p<0.001), and antibiotic administration (4.2%-84.0%, p<0.001). No parameter displayed a decrease in compliance in the second PDSA cycle. The usage rate of the proforma for the second cycle was 82.9% across a total of 35 patients.

## Discussion

Detailed, precise, and understandable medical documentation as recommended by The Royal College of Surgeons of England is crucial to achieving good medical practice [10]. With trauma and orthopedics being the top surgical specialty in terms of litigation claims in the United Kingdom, good documentation is essential, especially in the perioperative period [12]. Our study demonstrates the marked improvement made in post-operative documentation by the use of a standardized proforma. Studies prior to this one have highlighted the benefits of using pre-templated checklists for both admission and operation documentation [7, 13-14]. The use of checklists in other specialties has had a significant effect on patient safety, especially in terms of minimizing potential errors [15]. This is further supported by the success of the WHO surgical safety checklist [16]. 

Prior to this project, our center utilized an operative note template, generally completed immediately after the procedure, and a freehand post-operative note written on day one. Our recommendation of the pre-templated proforma for the post-operative period has since replaced the latter. The pre-filled text with associated tick boxes improves the efficiency of documentation and acts as an aide memoire for staff to prompt aspects of the patient review. The prefilled text can also mitigate against poor handwriting which can slow down reviews of notes in the future. The proforma was designed to include information expected from national clinical guidelines. We believe the use of a proforma act to close the communication loop and allows a safe continuation of care. As such, this proforma can be easily transferred digitally and saved as an electronic template.

Despite the significant results shown in this study, we acknowledge further research is required, focusing on developing a template for specific orthopedic procedures. This would allow comparison between sub-specialties, also comparing freehand to pre-templated notes. Furthermore, results from repeated cycles would be valuable, with an increased sample size and assessment of compliance over a longer period of time. The use of a similar proforma in other orthopedic units would allow inter-institutional comparison and may provide a more comprehensive interpretation of results. 

## Conclusions

Documentation across multiple domains significantly improved following the implementation of the standardized proforma. This allowed insurance of a rhythmic flow of information from instruction to completion of the patient’s post-operative journey. The results of this study may promote the implementation of similar proformas in other units, and allow inter-institutional comparison.
